# Prescription of Anticholinergics in Tardive Syndromes: A “Dual Center” Survey among Psychiatrists

**DOI:** 10.1155/2020/8870945

**Published:** 2020-11-22

**Authors:** Anna Cutino, Roongroj Bhidayasiri, Carlo Colosimo

**Affiliations:** ^1^Department of Neurology, Santa Maria University Hospital, Terni, Italy; ^2^Chulalongkorn Centre of Excellence for Parkinson's Disease & Related Disorders, Department of Medicine, Faculty of Medicine, Chulalongkorn University and King Chulalongkorn Memorial Hospital, Thai Red Cross Society, Bangkok, Thailand; ^3^The Academy of Science, The Royal Society of Thailand, Bangkok, Thailand

## Abstract

**Methods:**

We assessed the attitude of two groups of psychiatrists (practicing in Italy and Thailand) towards the prescription of anticholinergics by a short online survey consisting of four questions. A total of one hundred questionnaires were sent out (50 in Italy and 50 in Thailand), and 42 psychiatrists responded to the survey.

**Results:**

When comparing the two cohorts, the difference, both for age and years of practice, was statistically significant (*p* < 0.00001 and *p* < 0.0001, respectively), with Thai psychiatrists being younger and with less time in practice as specialists. The results from the survey showed that the prescription of anticholinergic drugs at the beginning of the antipsychotic treatment was used by 5 psychiatrists (20.0%) of the Italian cohort and by 1 (5.9%) of the Thai cohort. Regarding the Italian psychiatrists who did not prescribe anticholinergics concomitantly with neuroleptics, we found that 5 (25.0%) of them had prescribed anticholinergics in the past but had abandoned this practice, while 15 (93.7%) of the Thai psychiatrists who did not prescribe anticholinergics at the moment of the survey answered that they had prescribed these drugs in the past.

**Conclusion:**

According to this preliminary survey, the practice to use anticholinergics as a treatment for tardive syndromes is still relatively common, particularly in psychiatrists of the older generation, but seemingly in decline over the years.

## 1. Introduction

Tardive syndromes are movement disorders resulting from chronic treatment with neuroleptics. The phenomenological spectrum of tardive syndromes is wide, including tardive dyskinesia or oro-bucco-lingual stereotypy, tardive akathisia, tardive dystonia, tardive tics, tardive tremor, tardive myoclonus, and drug-induced parkinsonism. The current diagnostic criteria for tardive syndromes include the following:

- Previous treatment with neuroleptics for at least three months
- Presence of involuntary movements of moderate severity, involving one or more body parts, or involuntary movements of mild intensity in two or more body parts
- Absence of other conditions that may cause involuntary movements [1]

Tardive syndromes are common, with an estimated prevalence of up to 39.7% in patients with long-term neuroleptics [2] and an incidence estimated of 4-5% per year [3]. General risk factors are old age, female gender, duration of neuroleptic therapy, and type of drug [4], with typical neuroleptic more liable than atypical ones. However, tardive dystonia, different from tardive dyskinesia, is more frequent in younger patients and in males [3].

The etiopathogenesis of these disorders is still poorly understood but probably involves plastic changes in the basal ganglia [5–9]. The most commonly accepted theory suggests a hypersensitivity of the postsynaptic dopaminergic neurons due to chronic dopaminergic blockage by antipsychotic drugs [5]. Other hypotheses focused on the role of glutamate [6], GABA [7], changes in the nucleus accumbens dynorphinergic synapses [8], and reduction of superoxide dismutase activity in the basal ganglia leading to oxidative stress [9].

Tardive syndromes are managed by a variety of specialists of whom the majority are probably psychiatrists. The treatment of tardive syndromes remains cumbersome, and once the first step of reducing or stopping neuroleptics is unsuccessful, symptomatic therapy may be considered. According to the most recent evidence-based guidelines by the American Academy of Neurology (AAN), clonazepam and Ginkgo Biloba (level B) and amantadine and tetrabenazine (level C) are the only drugs with recognized symptomatic efficacy on tardive disorders [10, 11]. Anticholinergics have been proposed in the therapy of tardive syndromes since the early 60 s [12], despite only anecdotal reports of a positive effect and a lack of sound evidence supporting their usefulness. This practice was based on an imperfect understanding of drug-induced movement disorders, which were collectively labeled by psychiatrists as “extrapyramidal syndromes.” The reasoning was that if drug-induced parkinsonism improves with concomitant use of anticholinergics, other “extrapyramidal” syndromes, including tardive dyskinesia, should also improve and even be prevented by this treatment.

Despite anecdotal reports, no efficacy was found in clinical trials testing biperiden, benztropine, chlorprothixene, and trihexyphenidyl as a treatment for tardive syndromes [10], and they can sometimes aggravate or even trigger the onset of these manifestations [13]. The only positive evidence of efficacy is the use of trihexyphenidyl in the treatment of tardive dystonia, demonstrating an improvement in up to 46% of the treated cases [14, 15]. Despite the general lack of evidence supporting the use of anticholinergics in the treatment of tardive syndromes, we felt that in routine clinical practice, they were still widely employed by psychiatrists when starting neuroleptic therapy, as a possible prophylactic or suppressive therapy for tardive dyskinesia and other tardive syndromes. Therefore, this study aimed to evaluate the attitude of psychiatrists from Italy and Thailand, as representatives of Western European and Eastern Asian countries, towards the use of anticholinergics to counteract tardive syndromes, being aware that differences in health care utilization, health insurance coverage, and socioeconomic factors could lead to practice differences.

## 2. Methods

We assessed the attitude of specialists in psychiatry towards the prescription of anticholinergics via a short survey. The survey was constructed by the senior authors who are experienced movement disorder neurologists with long experience in the management of tardive syndromes (RB and CC) during a face-to-face meeting, to gather evidence about the current prescribing behavior of the specialists who were contacted, but short enough to maximize the response rate. The physicians who took part in the survey were all board-certified psychiatrists, distributed into two cohorts. The first was composed of psychiatrists from Italy, and the second was composed of psychiatrists from Thailand. They received the questionnaire as a Word document attached to an e-mail and they answered it anonymously, recording only age, sex, and years of clinical practice as a specialist. The questionnaire took around 5 to 10 minutes to complete. Two weeks later, an e-mail reminder was sent. No award or compensation was offered to responders.

The questionnaire was composed of four questions:

- Are you using anticholinergics when you start a new patient with neuroleptics?
- Are you using anticholinergics also when you start atypical neuroleptics?
- Do you think is there any scientific evidence justifying the use of anticholinergics to treat or prevent tardive dyskinesia?
- Did you use anticholinergics for this purpose in the past? Or, have you never administered anticholinergics?

If the answer to the first question was Yes, the respondent was required to answer the second and the third questions; if the answer was No, the respondent was required to answer the last two questions. The questionnaire was designed to investigate the use of primary anticholinergics (e.g., trihexyphenidyl) and not other drug classes with anticholinergic properties. Data analysis was performed using the Mann–Whitney *U* test, comparing the demographic data of the two cohorts of the study, and Pearson's chi-square test for the questionnaire variables. A *p* value <0.05 was considered statistically significant.

## 3. Results

A total of one hundred questionnaires were sent out (50 in Italy and 50 in Thailand), and 42 psychiatrists responded to the survey, with a response rate of 42%. The responders included 25 psychiatrists from Italy and 17 psychiatrists from Thailand. The Italian psychiatrists were recruited from the central regions of Italy (6 from Rome province, 9 from Terni province, 4 from Perugia province, and 6 from Cagliari province). Thai psychiatrists were all working in the Bangkok conurbation and central region of Thailand. The mean (±SD) age of the psychiatrists who responded was 42.1 (±13.1) years, while the mean duration of their practice as specialists was 12.2 (±11.7) years. When analyzed as separate cohorts, Italian or Thai respondents, there was a significant difference in both age and work duration (Table 1). The mean age of Italian psychiatrists was 50.4 (±10.7) years, while the mean age of Thai psychiatrists was 30.2 (±4.1) years. The mean years in the practice of Italian psychiatrists were 18.9 (±11.6) years, while the mean years in the practice of Thai psychiatrists were 4.0 (±3.8) years. When comparing the two cohorts, the difference, both for age and years of practice, was statistically significant (*p* < 0.00001 and *p* < 0.0001, respectively).

Analysis of the responses given by the psychiatrists showed that the prescription of anticholinergics at the beginning of the antipsychotic treatment was used by 5 psychiatrists (20.0%) of the Italian cohort and by 1 (5.9%) of the Thai cohort, while 20 psychiatrists (80%) of Italian cohort and 16 (94.1%) of the Thai cohort did not follow with this clinical practice. However, the difference between the two cohorts was not significant (*p* = 0.19 Pearson and *p* = 0.40 Yates).

Regarding the group of Italian psychiatrists who answered positively to the first question, 4 of them (80%) also prescribed anticholinergics with atypical neuroleptics, and a similar number agreed with the opinion that there is scientific evidence about anticholinergic efficacy in the treatment of tardive syndromes. Only 1 (20%) of Italian psychiatrists from this group did not use anticholinergics as a treatment of tardive syndrome with atypical neuroleptics, while 1 (20%) did not reply to the question about the scientific evidence of this clinical practice. The Thai psychiatrist, who answered positively to the first question, answered negatively to the other two. The difference between the two cohorts was also not significant (*p* = 0.12 Pearson and *p* = 0.69 Yates).

Regarding the Italian psychiatrists who did not prescribe anticholinergics concomitantly with neuroleptics, we found that 5 of them (25.0%) had prescribed anticholinergics in the past but had abandoned this clinical practice, while the remaining 15 (75.0%) had never used it at the beginning of antipsychotic therapy. However, 6 of them (30.0%) declared, via a comment in the questionnaire, that they tended to prescribe these drugs when tardive dyskinesia appeared. On the contrary, 15 (93.7%) of the Thai psychiatrists who did not prescribe anticholinergics at the beginning of the antipsychotic therapy answered that they had prescribed anticholinergics in the past, and only 1 (6.3%) answered that he never used it. At this time, the difference between the two cohorts was statistically significant (*p* < 0.0001 Pearson and *p* = 0.0002 Yates).

We also compared the responses from Italian psychiatrists with less than, or more than, 20 years of clinical practice in this field (junior and senior groups, respectively). The first group contained 12 psychiatrists (48.0%) of the Italian cohort, and the second contained the remaining 13 (52.0%). The results showed that 1 (8.3%) of the juniors used anticholinergics at the beginning of the neuroleptic treatment versus 4 (30.8%) of the seniors. Four (36.4%) of the juniors abandoned the prescription of anticholinergics, versus 1 (11.1%) of the seniors. Eight psychiatrists (61.5%) of seniors had never used anticholinergics, versus 7 (63.6%) of the juniors; finally, 5 (38.5%) of the seniors only prescribed anticholinergics when tardive dyskinesia appeared, versus only 1 (8.3%) of the juniors.

Because of the young age of the Thai cohort as a whole, a further comparison was made between Thai psychiatrists and the group of Italian psychiatrists with less than 20 years of clinical practice. The results showed that 1 (8.3%) of the Italian cohort, versus 1 (5.9%) of the Thai one, still used anticholinergics at the beginning of an antipsychotic therapy, whereas 4 (33.3%) of the Italian cohort had abandoned the prescription of anticholinergics, versus 16 (94.1%) of the Thai cohort (*p* < 0.01). As a final point, 7 (58.3) of the Italian cohort had never used anticholinergics, against 1 (5.9%) of the Thai cohort (*p* < 0.001).

## 4. Discussion

The findings from our survey showed that differences in the use of anticholinergics in clinical practice are found amongst psychiatrists from Italy and Thailand. Indeed, curative or even “preventive” anticholinergic therapy for tardive syndromes is still a relatively common practice amongst Italian psychiatrists but seemingly in sharp decline. When comparing the two groups of Italian psychiatrists divided according to the years of practice, prescription of anticholinergics as a preventive measure or at the appearance of tardive symptoms seems a common clinical procedure by senior psychiatrists, while it decreases significantly with the reduction of years of practice. Conversely, the preventive use of anticholinergics is a rare habit amongst Thai psychiatrists, despite having been used in the past. We suppose that this may have been influenced by the relatively young age of the latter sample of psychiatrists, who on average had only a few years of clinical practice as specialists; however, other factors—such as different curricula and practices—cannot be excluded.

Prevention of tardive syndromes is crucial, but evidence supporting various approaches is lacking. Primary prevention requires, in the first place, physicians to carefully consider if neuroleptics are medically indicated for their patients, following the national and international guidelines on the management of psychotic disorders. Once treatment is started, regular evaluation to see if these antipsychotic medications need to be continued is essential. If discontinuation is not possible, general recommendations include the use of the lowest possible dose with a periodic reevaluation to prevent the development of tardive syndromes [10]. Conversely, evidence on secondary prevention is inconclusive, and this may lead physicians to consider using certain medications, such as anticholinergics.

Our study is limited by the lack of matched participants (concerning both the age and the years of practice) and the relatively low number of respondents, which is in line with similar web-based surveys in the literature [16]. Survey research in healthcare is an important tool to collect information about healthcare delivery, service use, and all issues related to the quality of care. Unfortunately, physicians are often a group with low survey response rates. However, looking at these limitations positively, they provide two different groups of participants where understanding the treatment of anticholinergics may differ due to several factors, including clinical experience, methods of training, updates on the current literature, and legal needs for continuing medical education. The differences in participant demographics may even provide critical information to us on which groups should be first targeted for education.

Besides, this type of survey is difficult to conduct, and the relatively low response rate of psychiatrists may have decreased the number of those who openly declare that they use anticholinergics, thus distorting the results because of a negative bias. Therefore, further data from both countries should be obtained to give a more accurate picture of how prevalent this therapeutic practice is. If the spread of the use of anticholinergics in psychiatry is then confirmed, scientific societies should proactively work to increase awareness on this topic, underlining the lack of evidence of this pharmacological practice (except for symptomatic treatment of tardive dystonia where anticholinergics may be considered) and the significant risk of side effects with anticholinergic use, especially in the elderly population [17]. In conclusion, the findings of our survey, although only preliminary and requiring confirmation by larger studies, highlight the need for education on evidence-based treatment of this common iatrogenic disorder.

## Figures and Tables

**Table 1:** Demographic data of the psychiatrists responding to the questionnaire.

	Age (years ± SD)	Time in clinical practice (years ± SD)
Italian cohort	50.4 (±10.68)	18.9 (±11.55)
Thai cohort	30.2 (±4.13)	4.0 (±3.81)
Both cohorts	42.7 (±13.12)	12.0 (±11.72)

## Data Availability

Data are available upon request to the corresponding author.

## References

[1] Schooler N. R., Kane J. M. (1982). Research diagnosis for tardive dyskinesia. *Archives of General Psychiatry*.

[2] Van Harten P. N., Matroos G. E., Hoek H. W., Kahn R. S. (1996). The prevalence of tardive dystonia, tardive dyskinesia, parkinsonism and akathisia the Curaçao extrapyramidal syndromes study: I. *Schizophrenia Research*.

[3] Kane J. M. (2004). Tardive dyskinesia rates with atypical antipsychotics in adults: prevalence and incidence. *The Journal of Clinical Psychiatry*.

[4] Jeste D. V. (2004). Tardive dyskinesia rates with atypical antipsychotic in older adults. *The Journal of Clinical Psychiatry*.

[5] Sethi K. D., Morgan J. C., Jankovic J., Tolosa E. (2007). Drug-induced movement disorders. *Drug Induced Movement Disorders*.

[6] Meshul C. K., Stallbaumer R. K., Taylor B., Janowsky A. (1994). Haloperidol-induced morphological changes in striatum are associated with glutamate synapses. *Brain Research*.

[7] Mazurek M. F., Rosebush P. I., Factor S. A., Lang A. E., Weiner W. J. (2005). Acute drug-induced dystonia. *Drug Induced Movement Disorders*.

[8] Meredith G. E., De Souza I. E. J., Hyde T. M., Tipper G., Wong M. L., Egan M. F. (2000). Persistent alterations in dendrites, spines, and dynorphinergic synapses in the nucleus accumbens shell of rats with neuroleptic-induced dyskinesias. *The Journal of Neuroscience*.

[9] Wu J. Q., Chen D. C., Tan Y. L., Soares J. C., Zhang X. Y. (2015). Mn-superoxide dismutase activity is associated with orofacial involuntary movements in schizophrenia patients with tardive dyskinesia. *Human Psychopharmacology: Clinical and Experimental*.

[10] Bhidayasiri R., Fahn S., Weiner W. J., Gronseth G. S., Sullivan K. L., Zesiewicz T. A. (2013). Evidence-based guideline: treatment of tardive syndromes: report of the guideline development subcommittee of the American academy of neurology. *Neurology*.

[11] Bhidayasiri R., Jitkritsadakul O., Friedman J. H., Fahn S. (2018). Updating the recommendations for treatment of tardive syndromes: a systematic review of new evidence and practical treatment algorithm. *Journal of the Neurological Sciences*.

[12] Ayd F. J. (1961). A survey of drug-induced extrapyramidal reactions. *Journal of the American Medical Association*.

[13] Lerner P. P., Miodownik C., Lerner V. (2015). Tardive dyskinesia (syndrome): current concept and modern approaches to its management. *Psychiatry and Clinical Neurosciences*.

[14] Kang U. J., Burke R. E., Fahn S. (1986). Natural history and treatment of tardive dystonia. *Movement Disorders*.

[15] Thenganatt M. A., Jankovic J. (2014). Treatment of dystonia. *Neurotherapeutics*.

[16] Cunningham C. T., Quan H., Hemmelgarn B. (2015). Exploring physician specialist response rates to web-based surveys. *BMC Medical Research Methodology*.

[17] Coupland C. A. C., Hill T., Dening T., Morriss R., Moore M., Hippisley-Cox J. (2019). Anticholinergic drug exposure and the risk of dementia. *JAMA Internal Medicine*.

